# Clinical Characteristics and Blood-Based Inflammatory Indices of Psychiatric Hospitalizations Among Children with Autism in China: A Retrospective Electronic Medical Records Study

**DOI:** 10.3390/brainsci16090903

**Published:** 2026-08-24

**Authors:** Chenxi Bao, Wenqing Li, Kangkang Chu, Qingxiang Liu, Ya Wang, Xiaoyan Ruan, Huimin Lü, Xi Liu, Xiaoyan Ke

**Affiliations:** Child Mental Health Research Center, The Affiliated Nanjing Brain Hospital, Nanjing Medical University, Nanjing 210029, China; arysa_bao@163.com (C.B.); liwenqing@stu.njmu.edu.cn (W.L.); drchkk@njmu.edu.cn (K.C.); nursenj@163.com (Q.L.); wy19830125@126.com (Y.W.); 15951613563@163.com (X.R.); 15996231078@163.com (H.L.); m13951737297@163.com (X.L.)

**Keywords:** ASD, psychiatric hospitalization, clinical characteristics, behavioral crises, inflammatory-related indices, systemic immune–inflammation index

## Abstract

**Highlights:**

**What are the main findings?**

- Aggression, psychosocial stressors, and ADHD-related symptoms were the leading drivers of psychiatric hospitalization in Chinese children with ASD, with markedly distinct admission patterns observed across intellectual disability status, sex, and developmental stage.
- Inpatient psychiatric treatment significantly reduced peripheral blood inflammatory indices (e.g., SII, WBC, NEU) and non-enzymatic antioxidant indicators, indicating measurable systemic biological changes following clinical intervention.

**What are the implications of the main findings?**

- Clinicians should adopt subgroup-specific inpatient assessment strategies, including awareness of catatonia in females (rare, 3/53, 5.7%), heightened vigilance for suicidality in adolescents, and ADHD-related crises in younger children, to enable developmentally tailored care.
- Routine blood-derived inflammatory indices may offer preliminary, accessible adjunctive information for understanding acute treatment-related biological changes in hospitalized autistic youth, though prospective validation with clinical outcome correlation is needed.

**Abstract:**

Background/Objectives: This study aimed to investigate the clinical characteristics associated with psychiatric hospitalizations in children with ASD and to evaluate changes in blood-based Inflammatory Indices following treatment. Methods: A retrospective study of 269 children and adolescents with ASD (≤18 years) admitted to Nanjing Brain Hospital between 2012 and 2023 was conducted. Electronic medical records were reviewed for demographic characteristics, primary reasons for hospitalization, ASD-specific clinical scale scores, and routine laboratory parameters. The systemic immune–inflammation index (SII), neutrophil-to-lymphocyte ratio (NLR), monocyte-to-lymphocyte ratio (MLR), platelet-to-lymphocyte ratio (PLR), and non-enzymatic antioxidant indicators (uric acid, total bilirubin, direct bilirubin, prealbumin) were calculated. Results: The mean age at admission was 12.13 years, and 80.3% of admitted patients were male. The leading reasons for hospitalization were aggression (76.21%), psychosocial stressors (48.32%), and ADHD-related symptoms (46.09%), with distinct patterns observed by intellectual disability status, sex, and developmental stage. Multiple regression analyses revealed that age, sex, and specific admission indications were significantly associated with baseline inflammatory indices (SII, NLR, PLR, WBC, NEU, PLT; all *p* < 0.05) and antioxidant indices (UA, TBIL, DBIL, PA; all *p* < 0.05). Among 196 patients with 4-week follow-up data, significant post-treatment reductions were observed in SII, WBC, NEU, UA, TBIL, and DBIL, alongside increased prealbumin (all *p* < 0.05). Conclusions: Psychiatric hospitalizations of Chinese children with ASD are driven by distinct behavioral and neurodevelopmental profiles. Concurrently, accessible blood-based inflammatory and antioxidant indices show significant baseline clinical associations and post-treatment alterations, suggesting their potential as adjunctive biological markers in acute psychiatric settings. These findings are limited by the retrospective single-center design, absence of a control group, and lack of post-treatment clinical severity assessments.

## 1. Introduction

Autism Spectrum Disorder (ASD) is a complex neurodevelopmental condition characterized by persistent deficits in social communication and social interaction, alongside restricted, repetitive patterns of behavior, interests, or activities [1]. Globally, ASD affects approximately 1 in 100 children, though prevalence estimates vary significantly across regions [2]. In the United States, the most recent surveillance data released in 2025 indicate that about 1 in 31 eight-year-old children have been identified with ASD, based on estimates from the Autism and Developmental Disabilities Monitoring (ADDM) Network—a marked increase compared with earlier reports [3]. In contrast, epidemiological studies in China have reported a lower estimated prevalence of 0.70% among children aged 6–12 years [4], based on DSM-5 diagnostic criteria with standardized clinical assessments (ADOS, ADI-R)**.** However, this difference may partly reflect methodological variations, including differences in assessment methodologies (direct clinical evaluation vs. surveillance-based record review), case ascertainment strategies, and evolving diagnostic awareness within the region.

Beyond the core diagnostic features of ASD, many children experience severe behavioral crises that may precipitate psychiatric hospitalization. These acute exacerbations, including aggression [5], self-injurious behavior (SIB) [6,7], emotion dysregulation [8], suicidality [9], catatonia [10] and severe obsessive symptoms [11], can require acute inpatient psychiatric management when safety or functional capacity is compromised. Aggression, whether physical, verbal, or combined, represents one of the most common pathways to hospitalization in this population, particularly when intensified by sensory overload or environmental stressors [5]. SIB, such as head-banging, biting, and skin-picking, may pose significant medical risk and often requires urgent clinical intervention [6]. Emotion dysregulation in ASD, characterized by heightened emotional reactivity and difficulties in returning to baseline, frequently manifests as severe behavioral outbursts that are difficult to manage in outpatient settings [8]. Suicidality, increasingly recognized among autistic youth, particularly during adolescence, is another critical indication for psychiatric admission [9]. Catatonia, although likely underrecognized in ASD, can present with marked psychomotor disturbance such as mutism, stupor, or exacerbated stereotypies and may require specialized inpatient evaluation [10]. Severe obsessive symptoms, including rigid adherence to routines resulting in functional impairment or aggressive resistance to interruption, often lead to hospitalization psychiatric care in ASD youth [11]. These symptoms often overlap with core ASD features, creating diagnostic complexity and escalating to crisis when compulsive rituals are disrupted [12]. Collectively, these behavioral crises highlight the complex interaction between core ASD characteristics and environmental demands, underscoring the need for intensive psychiatric care during periods of acute deterioration.

In line with increasing awareness of behavioral crises requiring hospitalization, studies have shown that children with ASD remain substantially more likely to require inpatient psychiatric care than their typically developing peers, with a more than sixfold increased risk of psychiatric admission [13,14]. Additionally, psychiatric healthcare costs for children with ASD are significantly higher than for children without ASD, particularly among those with co-occurring intellectual disabilities (ID) [15]. Caregiver-reported risk factors further indicate that single-parent households, later age at diagnosis, and self-injurious and aggressive behaviors significantly elevate the likelihood of hospitalization [16]. Co-occurring affective symptoms, including depression and obsessive–compulsive symptomatology, may further exacerbate emotional and behavioral instability, increasing the need for intensive psychiatric management [17]. Early identification and timely intervention targeting these high-risk features are therefore critical for preventing acute clinical deterioration and reducing hospitalization burden. Hospitalization patterns among children with ASD also demonstrate substantial heterogeneity. Clinical presentations vary by developmental stage, sex, cognitive functioning, and symptom severity [13]. Sex-based differences have also been observed, with females tending to exhibit more internalizing symptoms and males more externalizing behavioral disturbances [18]. Additionally, individuals with co-occurring ID often show greater functional impairment and vulnerability to severe behavioral crises, contributing to distinct hospitalization profiles [19].

However, most existing evidence regarding psychiatric hospitalization in ASD has been derived from Western populations, and large-scale data from China remain limited. A recent study [20] based on the same clinical database examined patterns of psychotropic medication use among hospitalized children with ASD in China, with particular attention to the role of ID. While that study focused on pharmacological management and prescription patterns, the current investigation constitutes an entirely distinct research component: it characterizes hospitalization triggers, clinical severity profiles, and—critically—integrates peripheral blood inflammatory and antioxidant indices as biological correlates of acute behavioral crises and treatment response. These distinct analytical objectives and outcome domains ensure that the two reports address non-overlapping scientific questions. Other research in China has focused primarily on healthcare service utilization at a systems level. For example, an analysis of hospital records in Beijing [21] examined patterns of medical service use and associated costs among individuals with ASD but did not specifically address psychiatric admissions or behavioral crisis management. Consequently, critical gaps persist in understanding the clinical characteristics, admission triggers, and treatment-related changes among hospitalized autistic children within China’s mental health service system. Real-world clinical studies leveraging electronic medical records (EMRs) are particularly scarce. EMR-based investigations provide comprehensive and objective clinical information derived from routine practice, including admission triggers, symptom severity assessments, treatment courses, and laboratory findings. Such data offer valuable opportunities to characterize hospitalization patterns, identify clinically relevant risk factors, and evaluate treatment-related changes in hospitalized children with ASD within real-world healthcare systems.

Emerging evidence suggests that immune dysregulation and systemic inflammatory responses may play important roles in the pathophysiology of ASD and its associated behavioral manifestations [22,23]. However, many commonly studied inflammatory biomarkers rely on specialized laboratory assays, limiting their feasibility in routine clinical practice. In recent years, peripheral blood-derived inflammatory indices—such as the neutrophil-to-lymphocyte ratio (NLR), platelet-to-lymphocyte ratio (PLR), monocyte-to-lymphocyte ratio (MLR), and systemic immune–inflammation index (SII)—have gained increasing attention as accessible and cost-effective biomarkers reflecting systemic inflammatory status [24]. These composite indices, derived from routine blood examinations, are considered more stable and clinically informative than single hematologic parameters. The systemic immune–inflammation index (SII) was selected because it integrates three cellular inflammatory components (platelets, neutrophils, lymphocytes) into a single composite metric, offering greater stability and sensitivity than individual ratios in reflecting systemic inflammatory burden. It is important to note that these peripheral blood-derived indices reflect systemic inflammatory status and do not directly measure central nervous system neuroinflammation. Although inflammatory abnormalities have been widely investigated in ASD, most studies have focused on community samples or case–control designs, with relatively limited attention to hospitalized pediatric populations experiencing acute behavioral crises. Furthermore, evidence regarding dynamic changes in inflammatory profiles during inpatient treatment remains scarce, particularly in real-world clinical settings in China. Understanding inflammation-related alterations in hospitalized autistic children may provide valuable insights into the biological correlates of symptom exacerbation and treatment response.

Therefore, this study aimed to characterize the clinical features of hospitalized children with ASD using EMR data from a large tertiary psychiatric center in China. Specifically, we sought to (1) describe demographic and clinical characteristics associated with hospitalization, (2) examine factors related to admission patterns and length of stay, and (3) evaluate treatment-related changes in peripheral blood-derived inflammation-related indices. By integrating real-world clinical and laboratory data, this study aims to provide a more comprehensive understanding of hospitalization patterns and potential biological correlates in children with ASD.

## 2. Materials and Methods

### 2.1. Study Design and Participants

This retrospective clinical study included all children and adolescents with ASD who were admitted for inpatient psychiatric care at the Child Mental Health Research Center (NCMHRC), Nanjing Brain Hospital, between January 2012 and May 2023. Diagnostic confirmation and hospitalization occurred at this single specialized tertiary center. The sample was derived from consecutive admissions during this period, ensuring comprehensive representation of inpatient ASD cases in this specialized mental health facility. Inclusion criteria included: (1) age ≤ 18 years at the time of admission; (2) primary or secondary diagnosis of autism spectrum disorder (ASD; ICD-10 code F84) confirmed by board-certified child psychiatrists (WHO, 1993). Patients with a hospitalization duration of less than 24 h were excluded because they typically did not undergo complete clinical assessments or laboratory evaluations, rendering their data insufficient for analyzing treatment-related changes. Patients discharged against medical advice (AMA) were excluded because their treatment was incomplete and follow-up laboratory data were usually unavailable, which would introduce selection bias and compromise the validity of pre–post comparisons. Additionally, patients with documented acute infectious diseases (e.g., pneumonia, urinary tract infection, upper respiratory tract infection) or active autoimmune diseases (e.g., systemic lupus erythematosus, juvenile idiopathic arthritis, autoimmune thyroiditis) at admission were excluded based on comprehensive review of admission notes, discharge diagnoses, and past medical history in the EMRs.

The diagnosis of ID was determined based on a comprehensive review of medical records, which included clinical history, narrative documentation and results from standardized cognitive assessments. Standardized cognitive assessments were conducted as part of routine clinical care. Among the 269 patients, 186 (69.15%) were evaluated using the Wechsler Intelligence Scale for Children (WISC-IV), 45 (16.73%) using Raven’s Progressive Matrices, and 31 (11.52%) using the Peabody Picture Vocabulary Test (PPVT). In 7 (2.60%) patients, direct testing was not feasible (e.g., in patients with severe communication challenges), so the diagnosis relied on clinical judgment supported by the above records. An IQ threshold of <70 was used to define ID, whereas individuals with borderline intellectual functioning (BIF, IQ 70–85) were assigned to the non-ID group. BIF is not classified as ID in either ICD-10 or DSM-5, and because the clinical diagnostic cutoff for ID remains at IQ < 70, we grouped BIF with the non-ID category for this analysis, consistent with prior ASD research [25].

A total of 269 hospitalized children and adolescents with ASD were included in the study. All physicians and nurses involved in collecting relevant information were child psychiatrists and psychiatric nurses trained by researchers. The collected demographic information included age, gender, body mass index (BMI), birth mode, gestational age at birth, whether rehabilitation was conducted prior to hospitalization, family history of mental illness, co-occurring ID, length of hospital stay, reasons for hospitalization, and scores on the Autism Behavior Checklist (ABC), Childhood Autism Rating Scale (CARS), Autism Diagnostic Interview-Revised (ADI-R), and Autism Diagnostic Observation Schedule (ADOS).

Eight patients (3.0%) had a documented comorbid epilepsy diagnosis. Because the retrospective EMR design did not permit reliable ascertainment of acute peri-ictal or post-ictal status at admission, these patients were not systematically excluded from baseline cross-sectional analyses; however, none had follow-up laboratory data and were therefore excluded from the paired pre–post comparisons (Table 4).

### 2.2. Blood Count Analysis and Ratio Calculation

Blood samples were collected under fasting conditions (≥8 h) via standard venipuncture. Routine blood count parameters were measured using the Mindray BC-6800 (2012–2021) and BC-7500CRP (2021–2023) hematology analyzers (Mindray Bio-Medical Electronics Co., Ltd., Shenzhen, China). Biochemical indicators were analyzed using the Mindray BS-800 (2012–2022) and BS-2800M (2022–2023) automatic clinical chemistry analyzers (Mindray Bio-Medical Electronics Co., Ltd., Shenzhen, China). Standard laboratory quality control procedures and cross-instrument calibration were performed to ensure consistent measurement performance during instrument upgrading. Blood routine and biochemical indicators were extracted from EMRs, including white blood cell count (WBC), neutrophil count (NEU), platelet count (PLT), uric acid (UA), total bilirubin (TBIL), direct bilirubin (DBIL), albumin (ALB), prealbumin (PA), alanine aminotransferase (ALT), and aspartate aminotransferase (AST). Notably, UA, TBIL, DBIL, and PA represent non-enzymatic antioxidant indicators that collectively account for a substantial proportion of plasma antioxidant capacity and have demonstrated utility as accessible biomarkers in neuropsychiatric populations [26]. Unlike specialized oxidative stress assays, these parameters are routinely available in clinical chemistry panels, enhancing real-world applicability. In addition, four systemic immune–inflammation indices were calculated:

The specific calculation formulas for these indices are as follows:

SII = Platelet count × Neutrophil count/Lymphocyte count

NLR = Neutrophil count/Lymphocyte count

MLR = Monocyte count/Lymphocyte count

PLR = Platelet count/Lymphocyte count

These derived indices (SII, NLR, MLR, PLR) were used as inflammation-related indices in subsequent analysis.

### 2.3. Statistical Analysis

All data were statistically analyzed using SPSS Statistics for Windows, Version 29.0 (IBM Corp., Armonk, NY, USA). All analyses were two-tailed tests, and the significance level was set at α = 0.05. Given the exploratory, hypothesis-generating nature of this retrospective EMR-based study and the absence of a predefined primary biomarker endpoint, no correction for multiple comparisons was implemented across all analyses. We acknowledge that this approach elevates the risk of Type I error; accordingly, findings should be interpreted as preliminary until replicated in confirmatory studies with prespecified outcomes. Measurement data were expressed as means ± SD, and count data were expressed as numbers (percentages). Analysis of variance (ANOVA), chi-square test, *t*-test, or Mann–Whitney U test was used to compare demographic and clinicopathological features among the groups. For categorical variables, the chi-square test was used when all expected cell frequencies were ≥5; Fisher’s exact test was applied for 2 × 2 tables when ≥20% of cells had expected frequencies < 5. For RxC tables where expected frequencies were extremely small (e.g., total cell count < 5), statistical testing was omitted in favor of descriptive reporting. Multiple linear regression models were used to assess the effects of age, gender, coexisting ID, and different reasons for hospitalization on clinical biomarkers. Prior to multiple linear regression analysis, assumptions were verified: multicollinearity was assessed using variance inflation factor (VIF), with values < 10 indicating acceptable levels; normality of residuals was examined using the Shapiro–Wilk test and Q–Q plots; and homoscedasticity and linearity were evaluated through residual scatter plots.

## 3. Results

### 3.1. Clinical Characteristics and Hospitalization Patterns

A total of 269 children and adolescents with ASD were included in the study. The average age at admission was 12.13 ± 3.28 years (range 4–18 years), with 80.3% being male (n = 216). The mean length of stay (LOS) was 38.37 ± 24.35 days (range 2–75 days).

**Figure 1:**
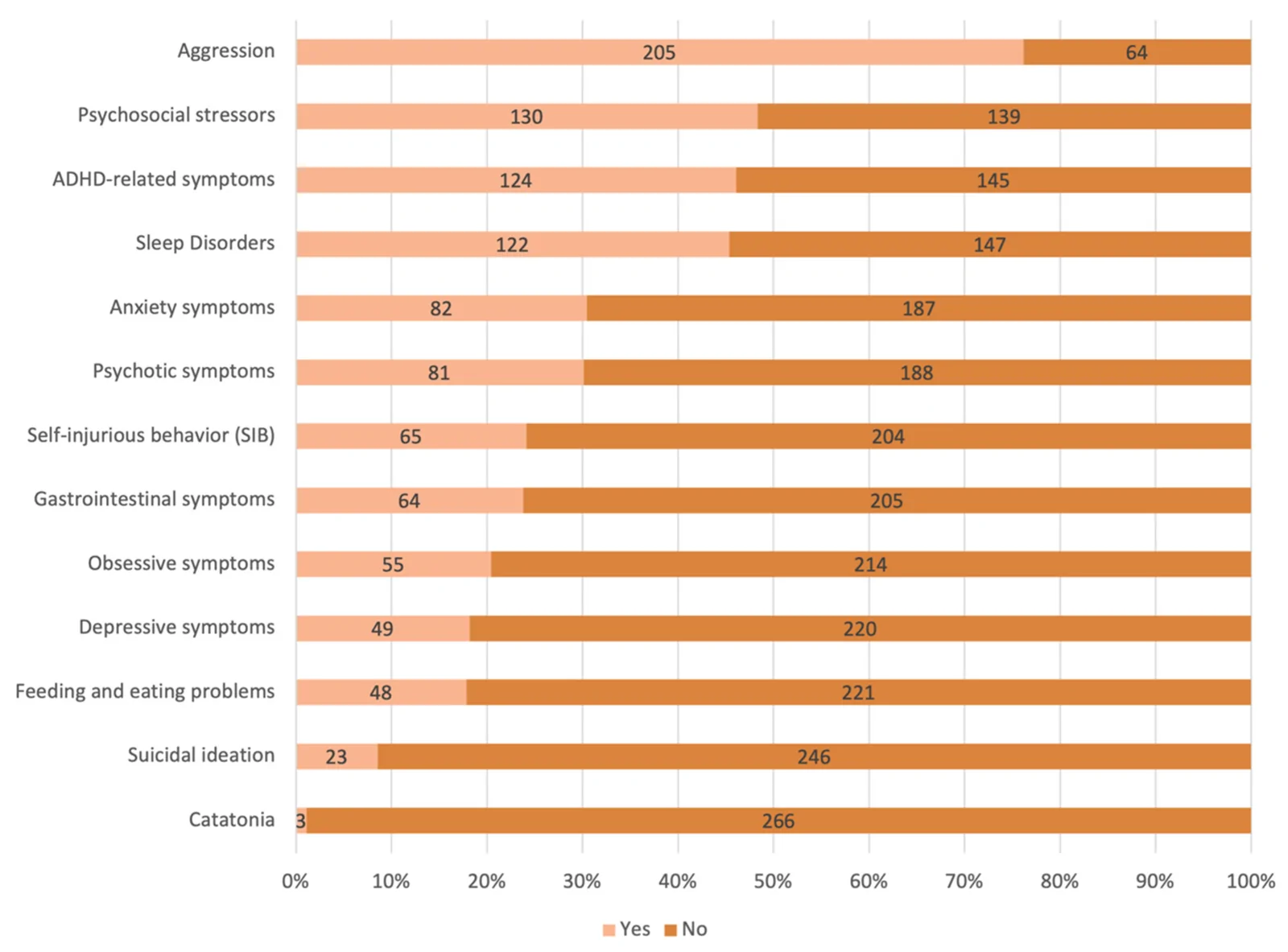
Most common causes of hospitalization for children with ASD between 2012 and 2023.

Hospitalization reasons were categorized based on a comprehensive review of EMRs and the previous literature, encompassing the following domains: psychosocial stressors, psychotic symptoms, catatonia, aggression, ADHD (attention-deficit/hyperactivity disorder)-related symptoms, depressive symptoms, anxiety symptoms, obsessive symptoms, self-injurious behavior (SIB), suicidal ideation, sleep disorders, feeding and eating problems, and gastrointestinal symptoms. The most prevalent primary reasons for admission were aggression (76.21%), psychosocial stressors (48.32%), and ADHD-related symptoms (46.09%). For more detailed results, please refer to Figure 1.

### 3.2. Subgroup Analyses by Intellectual Disability, Gender, and Age

Patients were stratified by intellectual functioning into two groups: ASD with co-occurring intellectual disability (ID:IQ ≤ 70, n = 114) and ASD without ID (IQ > 70, n = 155). Children with ID were more likely to have received pre-hospitalization rehabilitation training (χ^2^ = 6.87, *p* = 0.009). These children exhibited more severe autism symptoms, as indicated by higher scores on the ABC, ADI-Communication, and ADOS-Total (t = 3.03, *p* = 0.03; t = 2.02, *p* = 0.04; t = 3.95, *p* < 0.001), but had shorter hospital stays (t = −2.19, *p* = 0.029). In contrast, children without ID were more likely to be hospitalized due to depressive symptoms (χ^2^ = 16.65, *p* < 0.001), psychosocial stressors (χ^2^ = 7.502, *p* = 0.006), and gastrointestinal symptoms (χ^2^ = 6.98, *p* = 0.008).

Gender-based differences were also observed. Male patients were more likely to have received pre-hospitalization rehabilitation training (χ^2^ = 5.54, *p* = 0.019) and to be hospitalized due to obsessive symptoms (χ^2^ = 6.75, *p* = 0.009), whereas female patients were more likely to be hospitalized due to catatonia (Fisher’s exact test, *p* = 0.007). Detailed data can be found in Table 1.

Age-stratified analyses revealed distinct hospitalization patterns. Younger children (≤6 years, n = 25) were more likely to be hospitalized due to ADHD-related symptoms (χ^2^ = 13.92, *p* < 0.001), had shorter hospital stays (F = 6.35, *p* = 0.002), and exhibited higher ADOS-Total scores (F = 3.74, *p* = 0.027) compared to adolescents. Adolescents (13–18 years, n = 150) were more likely to be hospitalized due to depressive symptoms (χ^2^ = 14.20, *p* < 0.001) and suicidal ideation (χ^2^ = 10.17, *p* = 0.006). Detailed data can be found in Table 2.

### 3.3. Inflammatory and Oxidative Stress Indices

This study included 269 patients with ASD, all of whom underwent laboratory tests upon admission. The tests included multiple clinical indicators: WBC, NEU, PLT, UA, TBIL, DBIL, ALB, PA, ALT, AST, SII, NLR, MLR, and PLR.

#### 3.3.1. Baseline Clinical Associations

Multiple linear regression analyses examined associations between demographic and clinical factors (age, sex, co-occurring ID, and hospitalization reasons) and index levels. Age, gender, ADHD-related symptoms and gastrointestinal symptoms were significantly associated with inflammatory indices (SII, NLR, PLR, WBC, NEU, and PLT; all *p* < 0.05). Age, gender, psychotic symptoms, and feeding and eating problems were significantly associated with non-enzymatic antioxidant indicators (UA, TBIL, DBIL, and PA; all *p* < 0.05).

Further analyses demonstrated that, among children with ASD, increasing age was significantly associated with lower levels of several inflammatory indices, including SII (β = −0.146), WBC (β = −0.152), NEU (β = −0.144), and PLT (β = −0.169). In contrast, age was positively associated with non-enzymatic antioxidant indicators, including UA (β = 0.228), TBIL (β = 0.214), DBIL (β = 0.234), and PA (β = 0.151).

Sex-stratified analyses showed that male patients were associated with lower inflammatory indices, including WBC (β = −0.133) and PLT (β = −0.159), but higher PA levels (β = 0.288).

Admissions related to psychotic symptoms were associated with elevated non-enzymatic antioxidant indicators, particularly TBIL (β = 0.153). Hospitalizations due to feeding and eating problems were associated with higher DBIL levels (β = 0.155). Hospitalization due to ADHD-related symptoms was associated with lower NLR (β = −0.149) and PLR (β = −0.152). In contrast, admissions involving gastrointestinal symptoms were associated with higher PLT levels (β = 0.231). Detailed statistical results are presented in Table 3, with additional analyses provided in the Supplementary Materials.

Sensitivity analyses excluding the 8 epilepsy patients (3.0%) confirmed that the direction and statistical significance of the primary composite inflammatory indices (SII, NLR, MLR, PLR) remained unchanged. For individual parameters, the associations of WBC (*p* = 0.048) and NEU (*p* = 0.029) with ADHD-related symptoms and of PA (*p* = 0.043) with psychotic symptoms became statistically significant, whereas the association of AST with feeding and eating problems became non-significant (*p* = 0.060).

#### 3.3.2. Post-Treatment Changes

Among the 269 hospitalized children with ASD, follow-up laboratory assessments at 4 weeks of treatment (28 ± 7 days) were available for 196 patients. Paired *t*-test analyses demonstrated significant pre–post treatment differences in multiple biomarkers, including inflammation-related indices (SII, WBC, NEU), non-enzymatic antioxidant indicators (UA, TBIL, DBIL, PA), and liver function parameters (ALT, AST) (all *p* < 0.05).

Compared with baseline levels, SII, WBC, NEU, UA, TBIL, DBIL, ALT, and AST decreased significantly after treatment, whereas PA levels increased significantly. As shown in Table 4, Cohen’s d effect sizes for these significant changes were predominantly small to medium (range: 0.154–0.375), suggesting modest clinical magnitude despite statistical significance. Detailed results can be found in Table 4.

## 4. Discussion

This study provides one of the first comprehensive characterizations of psychiatric hospitalizations among children and adolescents with ASD in China, integrating real-world EMR data with blood-based inflammatory indices in a large tertiary psychiatric center. Our findings demonstrate that acute behavioral crises, including aggression, psychosocial stressors, and ADHD-related symptoms, constitute the predominant drivers of hospitalization in this population. Distinct hospitalization patterns emerged across intellectual functioning, sex, and developmental stage. Importantly, these findings align with prior reports from Western settings, suggesting cross-cultural consistency in the primary triggers of inpatient care for autistic youth.

In our cohort, aggression (76.2%) was the predominant reason for psychiatric admission, consistent with findings from Western populations where externalizing behaviors may warrant inpatient intervention [13,16]. However, the prominence of psychosocial stressors (48.32%), including acute family conflicts, school-related crises, and caregiver burden, may reflect distinctive help-seeking patterns in Chinese families. In this context, hospitalization is often perceived as the primary avenue for managing severe behavioral escalations, particularly given the limited availability of community-based crisis intervention services and 24-h professional support [27,28]. The substantial proportion of ADHD-related symptoms (46.09%) further underscores the clinical complexity of this population, as attention-deficit/hyperactivity disorder frequently co-occurs with ASD and exacerbates behavioral dysregulation [29].

Marked heterogeneity in hospitalization patterns was observed across intellectual functioning, sex, and developmental stage. Children with co-occurring ID exhibited more severe core autism symptoms yet experienced shorter hospital stays. This apparent paradox may reflect multiple contributing factors: greater diagnostic clarity facilitating rapid intervention [30], limited family resources for extended hospitalization, or more rapid behavioral stabilization in structured inpatient environments [31]. Specifically, patients with more profound impairments are often transferred earlier from the acute psychiatric ward to external specialized long-term care or rehabilitation facilities, which further curtails the duration of acute inpatient admission. ID was more frequently observed among catatonia-related admissions, although this association did not reach statistical significance after applying Fisher’s exact test (*p* = 0.075) due to the very low cell counts. The higher proportion of children with ID receiving pre-hospitalization rehabilitation training likely reflects greater prior engagement with developmental services, a pattern also observed in our previous analysis of this cohort [20]. Conversely, children without ID were more frequently hospitalized for depressive symptoms, psychosocial stressors, and gastrointestinal symptoms, highlighting the need for affective monitoring and somatic symptom management in higher-functioning autistic youth [32,33].

Sex-based differences revealed distinct hospitalization presentations, with males more frequently admitted for obsessive symptoms and females disproportionately for catatonia. This pattern may reflect diagnostic delays and compensation-related masking in females with ASD, leading to more severe crisis with presentation of psychomotor disturbances at first hospitalization [34], rather than a true sex difference in catatonia prevalence. The distinctive severity profile observed among female inpatients warrants increased clinical vigilance for catatonic symptoms in this subgroup. Age-stratified analyses revealed developmental-stage-specific patterns that underscore the heterogeneity of psychiatric hospitalization in ASD. Young children (≤6 years) were most frequently hospitalized for ADHD-related symptoms, had the shortest lengths of stay, and exhibited higher ADOS total scores, reflecting the early emergence of hyperactivity and core autistic features that may prompt acute intervention [35]. In contrast, adolescents (13–18 years) were more likely to be hospitalized for depressive symptoms and suicidal ideation (13.3% vs. 0% in younger children)—consistent with the well-documented rise in internalizing disorders and suicide risk during adolescence in ASD [9], when social–cognitive maturation and emerging comorbidities converge to elevate crisis risk [34]. These findings emphasize that psychiatric hospitalization in ASD is not a uniform clinical entity but instead reflects developmental and neuropsychological diversity, warranting developmentally tailored assessment and intervention strategies across the pediatric age spectrum.

A key innovation of this study is the integration of inflammation-related indices and non-enzymatic antioxidant indicators into the characterization of hospitalized children with ASD. At baseline, increasing age was associated with lower inflammatory-related indices (SII, WBC, NEU, PLT) but higher non-enzymatic antioxidant indicators. This pattern is consistent with the physiological maturation of the immune system in neurotypical children, in whom baseline inflammatory tone naturally declines from early childhood through adolescence [36]. Due to the absence of a control group in our study, we cannot determine whether this age-related decline is ASD-specific or reflects normative developmental trajectories [24]. Male sex was associated with lower WBC and PLT but higher PA levels, indicating sex-specific differences in inflammatory tone that may underlie differential behavioral presentations. Importantly, specific clinical presentations were linked to distinct biomarker profiles, offering preliminary support for biologically informed subtyping of ASD-related psychiatric crises. ADHD-related symptoms were associated with lower NLR and PLR—a finding that contrasts with the elevated inflammatory markers typically reported in community ADHD samples [37]. One possible interpretation is that behavioral dysregulation in ADHD-related presentations may be less strongly linked to acute peripheral inflammatory activation compared to other symptom domains in hospitalized autistic youth, or that prior treatment effects may have modulated inflammatory profiles prior to admission. Conversely, gastrointestinal symptoms were associated with higher platelet counts, consistent with prior reports of systemic inflammation accompanying gastrointestinal disturbances in ASD populations [38]. This observational association does not, however, provide direct evidence for gut–brain axis mechanisms in our cohort. Psychotic symptoms and feeding and eating problems were associated with elevated non-enzymatic antioxidant indicators (TBIL, DBIL), potentially reflecting compensatory upregulation of antioxidant defenses in response to oxidative stress, a mechanism implicated in psychotic disorders [26].

Following four weeks of inpatient treatment, significant reductions were observed in inflammatory-related indices (SII, WBC, NEU) and non-enzymatic antioxidant indicators (UA, TBIL, DBIL, ALT, AST), alongside increased PA. Notably, SII decreased significantly, whereas PLR remained stable, suggesting that the observed SII reduction was driven primarily by decreases in neutrophil and platelet components rather than lymphocyte dynamics. Notably, the effect sizes for these changes were predominantly small to medium (Cohen’s d < 0.4 for most markers), indicating that the observed statistical differences, while consistent, may not represent clinically substantial biological shifts. Concurrently, the decrease in liver enzymes (ALT, AST) suggests resolution of stress-related hepatic dysfunction, and the increase in PA indicates nutritional recovery. The concurrent decrease in ALT and AST may reflect both resolution of acute stress-related hepatic dysfunction and recovery from potential medication-induced hepatotoxicity, as psychotropic medications were commonly administered during hospitalization. Without a control group or detailed medication timing data, these mechanisms cannot be disentangled in this observational design. These post-treatment changes may reflect multiple converging mechanisms: (a) resolution of acute stress-related neuroimmune activation following behavioral crisis stabilization [39]; (b) improved nutritional and metabolic status as indicated by prealbumin recovery [40]; and (c) resolution of intercurrent medical issues (e.g., gastrointestinal disturbances) that may have contributed to both behavioral exacerbation and inflammatory elevation [41]. Notably, NLR and PLR did not change significantly, suggesting that these composite indices may be less sensitive to short-term treatment effects in this population. The persistence of elevated NLR and PLR has been associated with chronic inflammation in ASD, and longer follow-up may be required to observe meaningful reductions. While causality cannot be inferred from this observational design, these findings suggest that peripheral inflammatory indices may serve as accessible biomarkers for monitoring treatment response in acute psychiatric settings. However, given the exploratory nature of this study and the absence of correction for multiple comparisons, these findings should be interpreted cautiously and require replication in confirmatory studies.

Our findings have several clinical implications. Given that aggression, ADHD-related symptoms, and psychosocial stressors are the predominant admission triggers, early identification and management of these risk factors in outpatient settings may help prevent hospitalization. Inpatient care should adopt subgroup-stratified strategies: clinicians should be alert to catatonic symptoms in females, depressive symptoms and suicidality in adolescents, and ADHD-related symptoms in young children. Additionally, the significant post-treatment changes in routine blood markers (e.g., SII, WBC, neutrophil counts) suggest that these peripheral inflammatory indices may serve as accessible, cost-effective adjunctive biomarkers for monitoring treatment response in hospitalized autistic youth. Compared with neuroimaging or genetic testing, these indices offer advantages in cost, accessibility, and clinical scalability.

Several limitations should be acknowledged. The retrospective, single-center design limits generalizability and precludes causal inference. The absence of a control group (e.g., non-hospitalized ASD outpatients, hospitalized non-ASD psychiatric patients, or healthy age-matched peers) precludes any causal attribution of biomarker changes to hospitalization, treatment, or ASD-specific processes. The observed pre–post changes may reflect natural temporal variation, regression to the mean, or nonspecific effects of institutionalization, independent of clinical intervention. Inflammatory indices derived from peripheral blood lack diagnostic specificity and reflect general physiological state rather than central nervous system inflammation. Follow-up laboratory assessments were available for only 72.9% of the cohort, representing substantial patient attrition. Patients lost to follow-up may have differed systematically from those with complete data (e.g., earlier discharge due to clinical improvement, transfer to specialized facilities, or clinical deterioration requiring alternative management), potentially affecting the generalizability of the post-treatment biomarker findings. A critical limitation is the absence of post-treatment clinical severity assessments. Discharge decisions in this clinical setting were based on multidisciplinary clinical consensus rather than standardized scale re-evaluation, precluding correlation between biomarker changes and objective behavioral improvement. This severely limits the clinical interpretability of the laboratory findings. Psychotropic medications (antipsychotics, mood stabilizers, antidepressants, and benzodiazepines) were commonly administered during hospitalization according to individualized clinical protocols, but specific agents, dosages, timing, and changes were not systematically recorded in a format permitting quantitative analysis. Consequently, the observed biomarker changes cannot be disentangled from potential pharmacological effects, and any biological interpretation remains speculative. Use of systemic corticosteroids within the preceding 4 weeks was not systematically documented in the EMRs and could not be reliably excluded, representing a potential unmeasured confounder for inflammatory indices. Additionally, eight patients (3.0%) had comorbid epilepsy. While acute peri-ictal states could not be reliably excluded at baseline, sensitivity analyses confirmed the robustness of the core inflammatory indices, and the absence of follow-up data in these patients left the longitudinal comparisons unaffected. Future prospective, multi-center studies with standardized biospecimen collection and comparison groups are needed to validate these findings and elucidate causal pathways linking immune dysregulation to behavioral decompensation.

## 5. Conclusions

This study characterizes psychiatric hospitalizations among children with ASD in a large Chinese tertiary center, demonstrating distinct clinical profiles by intellectual disability, sex, and developmental stage. Routine blood-based inflammatory markers are associated with specific clinical presentations and change significantly during inpatient treatment. These findings highlight clinical heterogeneity and suggest that accessible biomarkers may aid in monitoring treatment response. Future prospective, multi-center studies with control groups are needed to validate these patterns and reduce preventable admissions.

## Figures and Tables

**Table 1:** Demographic and clinical characteristics by intellectual disability status and sex.

Variables	ID Group		t/χ^2^	Gender Group		t/χ^2^
	ID n = 114	Non-ID n = 155		Male n = 216	Female n = 53	
Age	11.71 ± 3.38	12.43 ± 3.19	−1.80	12.25 ± 3.22	11.62 ± 3.54	−1.25
Gender	94(82.5%)	122(78.7%)	0.58	-	-	-
Gestational age at birth	106(93.0%)	145(93.5%)	0.03	201(93.1%)	50(94.3%)	0.11
Rehabilitation training	23(20.2%)	14(9%)	6.87 *	35(16.2%)	2(3.8%)	5.54 *
Family history	27(23.7%)	40(25.8%)	0.15	52(24.1%)	15(28.3%)	0.40
Intellectual Disability	-	-	-	94(43.5%)	20(37.7%)	0.58
Length of stay	34.59 ± 21.78	41.14 ± 25.79	−2.19 *	38.06 ± 25.03	39.64 ± 21.52	−1.70
ABC	49.38 ± 25.79	34.92 ± 26.05	3.03 *	41.76 ± 29.17	37.52 ± 23.65	−0.67
CARS	31.92 ± 5.74	31.67 ± 2.82	0.34	31.70 ± 4.53	32.03 ± 2.96	0.36
ADI-Socialization	15.55 ± 6.81	14.01 ± 6.47	1.34	14.67 ± 7.00	14.24 ± 5.23	−0.33
ADI-Communication	9.78 ± 5.00	8.10 ± 4.62	2.02 *	8.92 ± 4.99	8.06 ± 4.21	−0.89
ADI-RSB	4.15 ± 2.63	4.13 ± 3.85	0.02	4.39 ± 3.62	3.33 ± 2.61	−1.55
ADOS-Total	15.15 ± 4.87	11.78 ± 3.96	3.95 **	13.08 ± 4.57	12.38 ± 4.53	−0.68
***Chief complaint***						
Psychosocial stressors	44(38.6%)	86(55.5%)	7.50 *	101(46.8%)	29(54.7%)	1.07
Psychotic symptoms	39(34.2%)	42(27.1%)	1.58	63(29.2%)	18(34.0%))	0.46
Catatonia	3(2.6%)	0(0.0%)	0.075 ^a^	0(0.0%)	3(5.7%)	0.007 ^a^
Aggression	89(78.1%)	116(74.8%))	0.37	167(77.3%)	38(71.7%)	0.74
ADHD-related symptoms	57(50.0%)	67(43.2%)	1.21	99(45.8%)	25(47.2%)	0.03
Depressive symptoms	8(7.0%)	41(26.5%)	16.65 **	35(16.2%)	14(26.4%)	2.97
Anxiety symptoms	30(26.3%)	52(33.5%)	1.62	69(31.9%)	13(24.5%)	1.10
Obsessive symptoms	24(21.1%)	31(20.0%)	0.04	51(23.6%)	4(7.5%)	6.75 *
Self-injurious behavior	27(23.7%)	38(24.5%)	0.02	50(23.1%)	15(28.3%)	0.61
Suicidal ideation	6(5.3%)	17(11.0%)	2.73	16(7.4%)	7(13.2%)	0.178 ^a^
Sleep Disorders	47(41.2%)	75(48.4%)	1.3	92(42.6%)	30(56.6%)	3.37
Feeding and eating problems	21(18.4%)	27(17.4%)	0.83	35(16.2%)	13(24.5%)	2.01
Gastrointestinal symptoms	18(15.8%)	46(29.7%)	6.98 *	49(22.7%)	15(28.3%)	0.74

ADI-RSB: ADI-Restricted and stereotyped behaviors. ADOS-Total: Total of communication and social interaction scores. Gender shows male (percentage); Gestational age at birth shows Full-term (percentage); Birth mode shows Natural delivery (percentage); * *p* < 0.05; ** *p* < 0.001; ^a^ = *p*-values from Fisher’s exact test.

**Table 2:** Demographic and clinical characteristics by age group.

Variables	Age Group			F/χ^2^
	≤6 n = 25	6–12 n = 94	13–18 n = 150	
Age	5.40 ± 0.76	10.15 ± 1.67	14.48 ± 1.40	543.67 **
Gender	19(76.0%)	74(78.7%)	123(82.0%)	0.71
Gestational age at birth	23(92.0%)	86(91.5%)	142(94.7%)	1.01
Rehabilitation training	7(28.0%)	14(14.9%)	16(10.7%)	5.58
Family history	9(36.0%)	28(29.8%)	30(20.0%)	4.77
Intellectual Disability	12(48.0%)	48(51.1%)	54(36.0%)	5.72
Length of stay	22.56 ± 14.85	38.45 ± 22.67	40.95 ± 25.72	6.35 *
ABC	54.17 ± 26.97	38.84 ± 30.97	39.29 ± 25.74	2.16
CARS	31.88 ± 7.32	31.49 ± 4.29	31.95 ± 3.22	0.17
ADI-Socialization	17.33 ± 8.94	14.31 ± 6.82	14.13 ± 5.88	1.94
ADI-Communication	9.57 ± 5.65	8.95 ± 5.50	8.43 ± 4.22	0.40
ADI-RSB	4.13 ± 3.87	4.29 ± 2.66	4.05 ± 3.78	0.07
ADOS-Total	15.23 ± 4.41	13.66 ± 5.08	11.95 ± 4.00	3.74 *
***Chief complaint***				
Psychosocial stressors	9(36.0%)	43(45.7%)	78(52.0%)	2.58
Psychotic symptoms	8(32.0%)	27(28.7%)	46(30.7%)	0.92
Catatonia	0(0.0%)	1(1.1%)	2(1.3%)	/
Aggression	21(84.0%)	74(78.7%)	110(73.3%)	1.84
ADHD-related symptoms	15(60.0%)	55(58.5%)	54(36.0%)	13.92 **
Depressive symptoms	1(4.0%)	9(9.6%)	39(26.0%)	14.20 **
Anxiety symptoms	5(20.0%)	30(31.9%)	47(31.3%)	1.43
Obsessive symptoms	4(16.0%)	19(20.2%)	32(21.3%)	0.38
Self-injurious behavior	5(20.0%)	18(19.1%)	42(28.0%)	2.73
Suicidal ideation	0(0.0%)	3(3.2%)	20(13.3%)	10.17 *
Sleep Disorders	6(24.0%)	43(45.7%)	73(48.7%)	5.27
Feeding and eating problems	4(16.0%)	17(18.1%)	27(18.0%)	0.06
Gastrointestinal symptoms	2(8.0%)	23(24.5%)	39(26.0%)	3.86

ADI-RSB: ADI-Restricted and stereotyped behaviors. ADOS-Total: Total of communication and social interaction scores. Gender shows male (percentage); Intellectual Disability shows IQ < 70; Gestational age at birth shows Full-term (percentage); Birth mode shows Natural delivery (percentage). Statistical comparison omitted for catatonia due to insufficient cell counts (n = 3). * *p* < 0.05; ** *p* < 0.001.

**Table 3:** The influence of various factors on blood markers.

Variables	Age	Gender	ID	Psychotic Symptoms	ADHD-Related Symptoms	Feeding and Eating Problems	Gastrointestinal Symptom
SII	0.027 *	0.770	0.665	0.358	0.800	0.803	0.829
NLR	0.108	0.553	0.655	0.632	0.027 *	0.947	0.204
MLR	0.954	0.523	0.897	0.719	0.316	0.879	0.551
PLR	0.087	0.608	0.544	0.587	0.022 *	0.093	0.606
WBC	0.019 *	0.040 *	0.151	0.768	0.113	0.089	0.843
NEU	0.030 *	0.457	0.295	0.941	0.057	0.356	0.540
PLT	0.008 *	0.012 *	0.297	0.189	0.139	0.737	0.002 *
UA	<0.001 **	0.002 *	0.150	0.697	0.257	0.477	0.190
TBIL	0.001 *	0.447	0.648	0.032 *	0.428	0.455	0.929
DBIL	<0.001 *	0.300	0.549	0.857	0.886	0.021 *	0.340
ALB	0.509	0.229	0.642	0.897	0.995	0.362	0.476
PA	0.020 *	<0.001 **	0.315	0.055	0.131	0.539	0.198
ALT	0.368	0.007 *	0.013*	0.991	0.520	0.205	0.099
AST	0.159	0.029 *	0.141	0.183	0.171	0.030 *	0.711

*p*-values are shown in the tables. * *p* < 0.05, ** *p* < 0.001. Multiple linear regression model was used to assess the impact of age, gender and various factors on inflammatory and oxidative stress markers. Complete regression outputs, including unstandardized coefficients (B), standard errors, and standardized coefficients (β), are provided in Supplementary Tables S1–S14. Table 3 presents a summary matrix of *p*-values for visual clarity.

**Table 4:** Comparison of Blood Parameters after treatment.

Variables	Baseline	4 Weeks	t	*p*	Cohen’s d	95% CI for Cohen’s d	
						Lower	Upper
SII	448.49 ± 318.05	392.82 ± 228.47	2.48	0.014 *	0.181	0.052	0.309
NLR	1.71 ± 1.20	1.52 ± 1.04	1.90	0.058	0.137	0.009	0.265
MLR	0.22 ± 0.12	0.25 ± 0.25	−1.53	0.064	−0.114	−0.242	0.014
PLR	120.42 ± 44.10	124.25 ± 52.76	−1.36	0.173	−0.089	−0.216	0.039
WBC	6.67 ± 1.90	5.96 ± 1.27	5.49	<0.001 **	0.375	0.244	0.506
NEU	3.58 ± 1.60	3.03 ± 0.99	4.92	<0.001 **	0.349	0.219	0.480
PLT	265.24 ± 62.78	263.89 ± 64.30	0.39	0.695	0.063	−0.064	0.190
UA	359.93 ± 94.81	330.92 ± 99.30	5.17	<0.001 **	0.340	0.206	0.473
TBIL	12.26 ± 8.75	9.45 ± 3.98	4.61	<0.001 **	0.288	0.156	0.419
DBIL	4.17 ± 2.14	3.46 ± 1.85	5.58	<0.001 **	0.307	0.175	0.439
ALB	44.92 ± 13.34	43.92 ± 3.90	1.04	0.296	0.070	−0.059	0.199
PA	219.21 ± 43.99	224.84 ± 38.44	−2.07	0.039 *	−0.156	−0.295	−0.016
ALT	20.41 ± 22.00	16.59 ± 16.15	3.39	<0.001 **	0.154	0.025	0.283
AST	21.97 ± 12.56	19.27 ± 11.68	3.42	<0.001 **	0.174	0.045	0.303

Baseline values represent the paired subset with complete 4-week follow-up laboratory data (n = 196); * *p* < 0.05, ** *p* < 0.001.

## Data Availability

The data presented in this study are available on request from the corresponding author due to privacy and ethical restrictions. Our study is a retrospective analysis of electronic medical records from children hospitalized with autism spectrum disorder. The dataset contains potentially identifiable patient health information, and its public sharing is restricted by the institutional ethics committee and data protection policies of Nanjing Brain Hospital. However, the data can be made available to qualified researchers upon reasonable request to the corresponding author (Prof. Xiaoyan Ke, email: kexynj@126.com), subject to appropriate data use agreements and ethical approval.

## Supplementary Materials

The following supporting information can be downloaded at: https://www.mdpi.com/article/10.3390/brainsci16090903/s1, Table S1: The influence of various factors on blood markers (dependent variable: SII); Table S2: The influence of various factors on blood markers (dependent variable: NLR); Table S3: The influence of various factors on blood markers (dependent variable: MLR); Table S4: The influence of various factors on blood markers (dependent variable: PLR); Table S5: The influence of various factors on blood markers (dependent variable: WBC); Table S6: The influence of various factors on blood markers (dependent variable: NEU); Table S7: The influence of various factors on blood markers (dependent variable: PLT); Table S8: The influence of various factors on blood markers (dependent variable: UA); Table S9: The influence of various factors on blood markers (dependent variable: TBIL); Table S10: The influence of various factors on blood markers (dependent variable: DBIL); Table S11: The influence of various factors on blood markers (dependent variable: ALB); Table S12: The influence of various factors on blood markers (dependent variable: PA); Table S13: The influence of various factors on blood markers (dependent variable: ALT); Table S14: The influence of various factors on blood markers (dependent variable: AST).
