# Supplementary material for: Clinical Characteristics and Blood-Based Inflammatory Indices of Psychiatric Hospitalizations Among Children with Autism in China: A Retrospective Electronic Medical Records Study
Source: Brain Sci. 2026 Aug 24;16(9):903. doi: 10.3390/brainsci16090903 (PMC13604248; doi:10.3390/brainsci16090903)
Supplement: Supplementary file 1 [file brainsci-16-00903-s001.zip › brainsci-4391408-supplementary.pdf]

Supplementary Files

Table S1: The influence of various factors on blood markers (dependent variable: SII)

Variables	Nonnormalized coefficient		Standardization coefficient	t	P
	B	Std.Error	Beta		
Age	-68.872	31.012	-0.146	-2.221	0.027*
Gender	-15.003	51.205	-0.019	-0.293	0.770
ID	17.959	41.390	0.028	0.434	0.665
Psychosocial stressors	-38.061	41.299	-0.061	-0.922	0.358
Psychotic symptoms	12.124	47.915	0.018	0.253	0.800
Catatonia	-122.822	192.819	-0.041	-0.637	0.525
Aggression	29.142	47.937	0.040	0.608	0.544
ADHD-related symptoms	-106.353	41.786	-0.170	-2.545	0.012*
Depressive symptoms	34.894	59.306	0.043	0.588	0.557
Anxiety symptoms	48.440	43.642	0.071	1.110	0.268
Obsessive symptoms	-7.527	50.539	-0.010	-0.149	0.882
Self-injurious behavior	-3.989	48.355	-0.005	-0.082	0.934
Suicidal ideation	8.465	78.754	0.008	0.107	0.914
Sleep Disorders	-21.050	46.714	-0.034	-0.451	0.653
Feeding and eating problems	11.628	54.064	0.014	0.215	0.830
Gastrointestinal symptoms	-11.844	54.933	-0.016	-0.216	0.829

*P<0.05, **P<0.001

Table S2: The influence of various factors on blood markers (dependent variable: NLR)

Variables	Nonnormalized coefficient		Standardization coefficient	t	P
	B	Std.Error	Beta		
Age	-0.183	0.113	-0.106	-1.612	0.108
Gender	0.111	0.187	0.039	0.594	0.553
ID	0.068	0.151	0.029	0.447	0.655
Psychosocial stressors	-0.035	0.151	-0.016	-0.234	0.815
Psychotic symptoms	0.084	0.175	0.034	0.479	0.632
Catatonia	-0.461	0.705	-0.042	-0.654	0.514
Aggression	0.066	0.175	0.025	0.374	0.708
ADHD-related symptoms	-0.340	0.153	-0.149	-2.227	0.027*
Depressive symptoms	0.153	0.217	0.052	0.706	0.481
Anxiety symptoms	0.130	0.160	0.052	0.812	0.417
Obsessive symptoms	-0.162	0.185	-0.057	-0.878	0.381
Self-injurious behavior	-0.099	0.177	-0.037	-0.561	0.575

Suicidal ideation	0.031	0.288	0.008	0.106	0.915
Sleep Disorders	-0.059	0.171	-0.026	-0.345	0.730
Feeding and eating problems	0.013	0.198	0.004	0.066	0.947
Gastrointestinal symptoms	-0.256	0.201	-0.096	-1.274	0.204

*P<0.05, **P<0.001

Table S3：The influence of various factors on blood markers (dependent variable: MLR)

Variables	Nonnormalized coefficient		Standardization coefficient	t	P
	B	Std.Error	Beta		
Age	-0.001	0.011	-0.004	-0.057	0.954
Gender	0.011	0.018	0.042	0.639	0.523
ID	-0.002	0.014	-0.009	-0.13	0.897
Psychosocial stressors	-0.005	0.014	-0.024	-0.361	0.719
Psychotic symptoms	0.017	0.017	0.072	1.004	0.316
Catatonia	-0.012	0.067	-0.012	-0.179	0.858
Aggression	0.028	0.017	0.112	1.688	0.093
ADHD-related symptoms	-0.021	0.015	-0.097	-1.433	0.153
Depressive symptoms	0.021	0.021	0.075	1.013	0.312
Anxiety symptoms	0.003	0.015	0.012	0.190	0.849
Obsessive symptoms	-0.002	0.018	-0.007	-0.108	0.914
Self-injurious behavior	-0.016	0.017	-0.066	-0.979	0.328
Suicidal ideation	-0.015	0.027	-0.038	-0.535	0.593
Sleep Disorders	0.003	0.016	0.015	0.196	0.845
Feeding and eating problems	-0.003	0.019	-0.010	-0.152	0.879
Gastrointestinal symptoms	-0.011	0.019	-0.045	-0.598	0.551

*P<0.05, **P<0.001

Table S4：The influence of various factors on blood markers (dependent variable: PLR)

Variables	Nonnormalized coefficient		Standardization coefficient	t	P
	B	Std.Error	Beta		
Age	-7.495	4.366	-0.111	-1.717	0.087
Gender	3.698	7.209	0.033	0.513	0.608
ID	3.537	5.827	0.039	0.607	0.544
Psychosocial stressors	-14.036	5.814	-0.158	-2.414	0.016
Psychotic symptoms	3.673	6.745	0.038	0.545	0.587
Catatonia	9.422	27.145	0.022	0.347	0.729
Aggression	3.408	6.748	0.033	0.505	0.614
ADHD-related symptoms	-13.563	5.883	-0.152	-2.306	0.022*
Depressive symptoms	7.240	8.349	0.063	0.867	0.387
Anxiety symptoms	7.555	6.144	0.078	1.230	0.220

Obsessive symptoms	-0.129	7.115	-0.001	-0.018	0.986
Self-injurious behavior	-2.189	6.807	-0.021	-0.322	0.748
Suicidal ideation	11.793	11.087	0.074	1.064	0.288
Sleep Disorders	-0.476	6.576	-0.005	-0.072	0.942
Feeding and eating problems	12.83	7.611	0.110	1.686	0.093
Gastrointestinal symptoms	3.994	7.733	0.038	0.516	0.606

*P<0.05, **P<0.001

Table S5：The influence of various factors on blood markers (dependent variable: WBC)

Variables	Nonnormalized coefficient		Standardization coefficient	t	P
	B	Std.Error	Beta		
Age	-0.438	0.186	-0.152	-2.356	0.019*
Gender	-0.635	0.307	-0.133	-2.066	0.040*
ID	0.357	0.248	0.093	1.439	0.151
Psychosocial stressors	0.304	0.248	0.080	1.227	0.221
Psychotic symptoms	-0.085	0.287	-0.021	-0.295	0.768
Catatonia	-1.393	1.157	-0.077	-1.204	0.230
Aggression	-0.021	0.288	-0.005	-0.074	0.941
ADHD-related symptoms	-0.399	0.251	-0.105	-1.591	0.113
Depressive symptoms	-0.289	0.356	-0.059	-0.813	0.417
Anxiety symptoms	0.037	0.262	0.009	0.142	0.887
Obsessive symptoms	0.013	0.303	0.003	0.043	0.965
Self-injurious behavior	0.101	0.290	0.023	0.348	0.728
Suicidal ideation	-0.673	0.472	-0.099	-1.425	0.155
Sleep Disorders	-0.211	0.28	-0.055	-0.752	0.453
Feeding and eating problems	-0.554	0.324	-0.112	-1.71	0.089
Gastrointestinal symptoms	0.065	0.33	0.015	0.199	0.843

*P<0.05, **P<0.001

Table S6：The influence of various factors on blood markers (dependent variable: NEU)

Variables	Nonnormalized coefficient		Standardization coefficient	t	P
	B	Std.Error	Beta		
Age	-0.358	0.164	-0.144	-2.186	0.030*
Gender	-0.202	0.271	-0.049	-0.745	0.457
ID	0.229	0.219	0.069	1.049	0.295
Psychosocial stressors	0.096	0.218	0.029	0.439	0.661
Psychotic symptoms	0.019	0.253	0.005	0.075	0.941
Catatonia	-1.080	1.019	-0.069	-1.059	0.291
Aggression	-0.009	0.253	-0.002	-0.036	0.971
ADHD-related symptoms	-0.422	0.221	-0.128	-1.912	0.057

Depressive symptoms	-0.050	0.314	-0.012	-0.159	0.873
Anxiety symptoms	0.075	0.231	0.021	0.324	0.746
Obsessive symptoms	-0.076	0.267	-0.019	-0.285	0.776
Self-injurious behavior	-0.011	0.256	-0.003	-0.042	0.967
Suicidal ideation	-0.236	0.416	-0.040	-0.568	0.571
Sleep Disorders	-0.191	0.247	-0.058	-0.775	0.439
Feeding and eating problems	-0.264	0.286	-0.061	-0.924	0.356
Gastrointestinal symptoms	-0.178	0.290	-0.046	-0.614	0.540

*P<0.05, **P<0.001

Table S7: The influence of various factors on blood markers (dependent variable: PLT)

Variables	Nonnormalized coefficient		Standardization coefficient	t	P
	B	Std.Error	Beta		
Age	-17.107	6.370	-0.169	-2.686	0.008*
Gender	-26.601	10.517	-0.159	-2.529	0.012*
ID	8.891	8.501	0.066	1.046	0.297
Psychosocial stressors	-14.611	8.483	-0.110	-1.722	0.086
Psychotic symptoms	-12.975	9.841	-0.089	-1.318	0.189
Catatonia	14.604	39.604	0.023	0.369	0.713
Aggression	5.140	9.846	0.033	0.522	0.602
ADHD-related symptoms	-12.727	8.583	-0.095	-1.483	0.139
Depressive symptoms	-2.412	12.181	-0.014	-0.198	0.843
Anxiety symptoms	8.735	8.964	0.060	0.975	0.331
Obsessive symptoms	14.264	10.380	0.086	1.374	0.171
Self-injurious behavior	10.017	9.932	0.064	1.009	0.314
Suicidal ideation	-4.443	16.175	-0.019	-0.275	0.784
Sleep Disorders	-7.914	9.595	-0.059	-0.825	0.410
Feeding and eating problems	3.739	11.104	0.022	0.337	0.737
Gastrointestinal symptoms	36.117	11.283	0.231	3.201	0.002*

*P<0.05, **P<0.001

Table S8: The influence of various factors on blood markers (dependent variable: UA)

Variables	Nonnormalized coefficient		Standardization coefficient	t	P
	B	Std.Error	Beta		
Age	33.931	9.555	0.228	3.551	<0.001**
Gender	48.890	15.455	0.203	3.163	0.002*
ID	18.165	12.593	0.093	1.443	0.150
Psychosocial stressors	-12.029	12.563	-0.062	-0.957	0.339
Psychotic symptoms	5.708	14.661	0.027	0.389	0.697
Catatonia	-12.342	57.194	-0.014	-0.216	0.829

Aggression	-0.909	14.896	-0.004	-0.061	0.951
ADHD-related symptoms	14.458	12.722	0.075	1.136	0.257
Depressive symptoms	-20.579	18.088	-0.083	-1.138	0.256
Anxiety symptoms	8.172	13.089	0.039	0.624	0.533
Obsessive symptoms	4.535	15.349	0.019	0.295	0.768
Self-injurious behavior	-6.254	14.678	-0.028	-0.426	0.670
Suicidal ideation	-18.87	24.195	-0.055	-0.780	0.436
Sleep Disorders	-7.756	14.391	-0.040	-0.539	0.590
Feeding and eating problems	11.641	16.337	0.047	0.713	0.477
Gastrointestinal symptoms	22.078	16.795	0.098	1.315	0.190

*P<0.05, **P<0.001

Table S9: The influence of various factors on blood markers (dependent variable: TBIL)

Variables	Nonnormalized coefficient		Standardization coefficient	t	P
	B	Std.Error	Beta		
Age	2.606	0.803	0.214	3.245	0.001*
Gender	-0.996	1.309	-0.050	-0.761	0.447
ID	-0.487	1.064	-0.030	-0.457	0.648
Psychosocial stressors	-1.527	1.060	-0.096	-1.441	0.151
Psychotic symptoms	2.657	1.231	0.153	2.158	0.032*
Catatonia	-0.614	4.856	-0.008	-0.126	0.900
Aggression	1.039	1.256	0.055	0.827	0.409
ADHD-related symptoms	-0.854	1.075	-0.054	-0.794	0.428
Depressive symptoms	-0.706	1.505	-0.035	-0.469	0.639
Anxiety symptoms	0.406	1.107	0.024	0.367	0.714
Obsessive symptoms	-0.303	1.300	-0.015	-0.233	0.816
Self-injurious behavior	-1.251	1.241	-0.068	-1.008	0.314
Suicidal ideation	-0.363	2.045	-0.013	-0.177	0.859
Sleep Disorders	0.635	1.211	0.040	0.524	0.601
Feeding and eating problems	1.036	1.385	0.050	0.748	0.455
Gastrointestinal symptoms	-0.127	1.421	-0.007	-0.089	0.929

*P<0.05, **P<0.001

Table S10: The influence of various factors on blood markers (dependent variable: DBIL)

Variables	Nonnormalized coefficient		Standardization coefficient	t	P
	B	Std.Error	Beta		
Age	0.739	0.208	0.234	3.553	<0.001**
Gender	0.352	0.339	0.068	1.040	0.300
ID	0.165	0.276	0.040	0.600	0.549
Psychosocial stressors	-0.447	0.274	-0.108	-1.628	0.105

Psychotic symptoms	0.273	0.319	0.061	0.857	0.393
Catatonia	1.408	1.257	0.073	1.120	0.264
Aggression	0.238	0.325	0.048	0.732	0.465
ADHD-related symptoms	0.040	0.278	0.010	0.143	0.886
Depressive symptoms	-0.069	0.390	-0.013	-0.178	0.859
Anxiety symptoms	0.179	0.287	0.040	0.626	0.532
Obsessive symptoms	0.045	0.337	0.009	0.135	0.893
Self-injurious behavior	-0.302	0.321	-0.063	-0.939	0.349
Suicidal ideation	-0.119	0.529	-0.016	-0.225	0.822
Sleep Disorders	-0.215	0.314	-0.052	-0.685	0.494
Feeding and eating problems	0.833	0.359	0.155	2.322	0.021*
Gastrointestinal symptoms	0.352	0.368	0.072	0.957	0.340

*P<0.05, **P<0.001

Table S11：The influence of various factors on blood markers (dependent variable: ALB)

Variables	Nonnormalized coefficient		Standardization coefficient	t	P
	B	Std.Error	Beta		
Age	-1.138	1.720	-0.042	-0.662	0.509
Gender	3.382	2.802	0.076	1.207	0.229
ID	1.061	2.278	0.029	0.466	0.642
Psychosocial stressors	2.291	2.269	0.064	1.010	0.314
Psychotic symptoms	-0.342	2.635	-0.009	-0.130	0.897
Catatonia	61.349	10.396	0.369	5.901	<0.001**
Aggression	-2.640	2.688	-0.062	-0.982	0.327
ADHD-related symptoms	0.014	2.300	0.000	0.006	0.995
Depressive symptoms	-2.162	3.222	-0.048	-0.671	0.503
Anxiety symptoms	2.720	2.369	0.071	1.148	0.252
Obsessive symptoms	-2.071	2.783	-0.047	-0.744	0.458
Self-injurious behavior	-2.900	2.658	-0.070	-1.091	0.276
Suicidal ideation	-0.125	4.378	-0.002	-0.029	0.977
Sleep Disorders	2.182	2.593	0.061	0.842	0.401
Feeding and eating problems	-2.707	2.965	-0.059	-0.913	0.362
Gastrointestinal symptoms	2.174	3.042	0.052	0.715	0.476

*P<0.05, **P<0.001

Table S12：The influence of various factors on blood markers (dependent variable: PA)

Variables	Nonnormalized coefficient		Standardization coefficient	t	P
	B	Std.Error	Beta		
Age	10.395	4.432	0.151	2.345	0.020*
Gender	32.123	7.115	0.288	4.515	<0.001**

ID	-5.903	5.863	-0.065	-1.007	0.315
Psychosocial stressors	-1.253	5.836	-0.014	-0.215	0.830
Psychotic symptoms	13.134	6.819	0.134	1.926	0.055
Catatonia	27.559	26.281	0.067	1.049	0.295
Aggression	-4.368	6.950	-0.041	-0.629	0.530
ADHD-related symptoms	8.945	5.909	0.099	1.514	0.131
Depressive symptoms	5.215	8.339	0.045	0.625	0.532
Anxiety symptoms	-5.844	6.054	-0.060	-0.965	0.335
Obsessive symptoms	-1.907	7.069	-0.017	-0.270	0.788
Self-injurious behavior	-14.600	6.791	-0.140	-2.150	0.033*
Suicidal ideation	21.767	11.132	0.137	1.955	0.052
Sleep Disorders	-1.485	6.690	-0.016	-0.222	0.825
Feeding and eating problems	4.648	7.553	0.040	0.615	0.539
Gastrointestinal symptoms	10.106	7.828	0.096	1.291	0.198

*P<0.05, **P<0.001

Table S13：The influence of various factors on blood markers (dependent variable: ALT)

Variables	Nonnormalized coefficient		Standardization coefficient	t	P
	B	Std.Error	Beta		
Age	1.870	2.073	0.058	0.902	0.368
Gender	9.217	3.377	0.174	2.730	0.007*
ID	6.855	2.733	0.160	2.509	0.013*
Psychosocial stressors	1.245	2.724	0.029	0.457	0.648
Psychotic symptoms	-0.037	3.159	-0.001	-0.012	0.991
Catatonia	-0.227	12.697	-0.001	-0.018	0.986
Aggression	4.046	3.198	0.081	1.265	0.207
ADHD-related symptoms	1.783	2.766	0.042	0.645	0.520
Depressive symptoms	-3.162	3.906	-0.058	-0.810	0.419
Anxiety symptoms	2.345	2.877	0.051	0.815	0.416
Obsessive symptoms	1.360	3.331	0.026	0.408	0.683
Self-injurious behavior	-2.800	3.184	-0.057	-0.880	0.380
Suicidal ideation	1.086	5.184	0.014	0.210	0.834
Sleep Disorders	2.625	3.096	0.062	0.848	0.397
Feeding and eating problems	-4.550	3.577	-0.083	-1.272	0.205
Gastrointestinal symptoms	6.021	3.634	0.121	1.657	0.099

*P<0.05, **P<0.001

Table S14：The influence of various factors on blood markers (dependent variable: AST)

Variables	Nonnormalized coefficient		Standardization coefficient	t	P
	B	Std.Error	Beta		

Age	-1.635	1.156	-0.092	-1.414	0.159
Gender	4.143	1.884	0.142	2.199	0.029*
ID	2.248	1.524	0.095	1.475	0.141
Psychosocial stressors	0.135	1.520	0.006	0.089	0.929
Psychotic symptoms	-2.353	1.762	-0.093	-1.335	0.183
Catatonia	-1.199	7.083	-0.011	-0.169	0.866
Aggression	-0.130	1.784	-0.005	-0.073	0.942
ADHD-related symptoms	-2.119	1.543	-0.091	-1.373	0.171
Depressive symptoms	-3.096	2.179	-0.103	-1.421	0.157
Anxiety symptoms	1.634	1.605	0.065	1.018	0.310
Obsessive symptoms	1.305	1.858	0.045	0.702	0.483
Self-injurious behavior	-0.964	1.776	-0.035	-0.543	0.588
Suicidal ideation	-0.358	2.892	-0.009	-0.124	0.901
Sleep Disorders	3.102	1.727	0.132	1.796	0.074
Feeding and eating problems	-4.357	1.995	-0.144	-2.184	0.030*
Gastrointestinal symptoms	0.751	2.027	0.027	0.371	0.711

*P<0.05, **P<0.001
